# Emergency Preparedness and Risk Communication Among African American Churches: Leveraging a Community-Based Participatory Research Partnership COVID-19 Initiative

**DOI:** 10.5888/pcd17.200408

**Published:** 2020-12-10

**Authors:** LaPrincess C. Brewer, Gladys B. Asiedu, Clarence Jones, Monisha Richard, Jamia Erickson, Jennifer Weis, Adeline Abbenyi, Tabetha A. Brockman, Irene G. Sia, Mark L. Wieland, Richard O. White, Chyke A. Doubeni

**Affiliations:** 1 Division of Preventive Cardiology, Department of Cardiovascular Medicine, Mayo Clinic, Rochester, Minnesota; 2 Center for Health Equity and Community Engagement Research, Mayo Clinic, Rochester, Minnesota; 3 Robert D. and Patricia E. Kern Center for the Science of Healthcare Delivery, Mayo Clinic, Rochester, Minnesota; 4 Hue-Man Partnership, Minneapolis, Minnesota; 5 Volunteers of America, Inc, Minneapolis, Minnesota; 6 Thrivent Financial, Inc, Rochester, Minnesota; 7 Center for Clinical and Translational Science, Mayo Clinic, Rochester, Minnesota; 8 Division of Infectious Diseases, Mayo Clinic, Rochester, Minnesota; 9 Division of Community Internal Medicine, Mayo Clinic, Rochester, Minnesota; 10 Division of Community Internal Medicine, Mayo Clinic, Jacksonville, Florida; 11 Department of Family Medicine, Mayo Clinic, Rochester, Minnesota

## Abstract

The coronavirus disease 2019 (COVID-19) crisis has disproportionately affected the African American population. To mitigate the disparities, we deployed an emergency preparedness strategy within an existing community-based participatory research (CBPR) partnership among African American churches to disseminate accurate COVID-19 information. We used the Centers for Disease Control and Prevention Crisis and Emergency Risk Communication framework to conduct a needs assessment, distribute emergency preparedness manuals, and deliver COVID-19–related messaging among African American churches via electronic communication platforms. A needs assessment showed that the top 3 church emergency resource needs were financial support, food and utilities, and COVID-19 health information. During an 8-week period (April 3–May 31, 2020), we equipped 120 churches with emergency preparedness manuals and delivered 230 messages via social media (Facebook) and email. For reach, we estimated that 6,539 unique persons viewed content on the Facebook page, and for engagement, we found 1,260 interactions (eg, likes, loves, comments, shares, video views, post clicks). Emails from community communication leaders reached an estimated 12,000 church members. CBPR partnerships can be effectively leveraged to promote emergency preparedness and communicate risk among under-resourced communities during a pandemic.

**Summary**

**What is already known on this topic?**

The coronavirus disease 2019 (COVID-19) pandemic has disproportionately affected African American communities with high infection and mortality rates; these high rates result from pre-existing chronic disease disparities and structural inequities.

**What is added by this report?**

A Centers for Disease Control and Prevention framework guided the implementation and evaluation of a COVID-19 emergency preparedness and risk communication strategy within an established community-based participatory research partnership with African American churches.

**What are the implications for public health practice?**

Leveraging existing infrastructure from academic–community partnerships can facilitate rapid dissemination of accurate COVID-19 health information and critical resources to address COVID-19–related inequities.

## Introduction

Partnerships with faith-based organizations are pivotal in rapidly engaging with racial/ethnic minority populations, who are often socially and economically marginalized and medically underserved, to address public health crises. Engagement with leaders of faith-based organizations on infectious disease prevention initiatives led to successes during the Ebola outbreak in West Africa and increases in influenza immunization rates during the H1N1 pandemic (1,2). African American communities have experienced disproportionately high novel coronavirus disease 2019 (COVID-19) infection and mortality rates, which have resulted from pre-existing chronic disease disparities and structural inequities (3–5). African American adults (27%) are more likely than Hispanic adults (13%) or White adults (13%) to personally know someone who has been hospitalized for or died of COVID-19 (6). Thus, community-engaged efforts are needed to mitigate the effect of COVID-19 among the African American population. From its inception, the Black church has consistently served as a resilient source to mobilize community members for capacity building to reach groups that have been marginalized and oppressed at the most troubling of times — especially in the areas of health and social injustice (7–9).

The FAITH! (Fostering African-American Improvement in Total Health) program is the first academic–community partnership between Mayo Clinic and local African American churches. It forged an authentic, community-based participatory research (CBPR) initiative to diminish chronic disease health disparities, particularly cardiovascular disease, in disproportionately affected African American communities (10). Since its founding in 2013 in Minnesota, FAITH! and its community partners have disseminated trusted health information and conducted research that has improved the health of local African American communities. Through community engagement and a social contextual framework, FAITH! has implemented innovative, community-driven programs, including signature mobile health lifestyle interventions (11). In 2017, FAITH! established a Community Steering Committee as a governing advisory board comprising diverse community advocacy and public health leaders to guide its programming (10).

In recognition of the COVID-19 pandemic’s detrimental effects on under-resourced communities of color and evidence of a disproportionate number of cases among African Americans, the Community Steering Committee repurposed itself from chronic disease prevention to immediately deploy strategies for a COVID-19 response. After the first diagnosis of COVID-19 in Minnesota in early March 2020 (12), the Community Steering Committee met on March 13, 2020, to brainstorm ideas for a practical emergency response plan within an established network of 120 African American churches representing more than 12,000 members throughout the Rochester and Minneapolis–St. Paul areas. Through mutual decision making, the Community Steering Committee prioritized providing churches with emergency preparedness plans and disseminating trusted health information. During the following week (March 16–20, 2020), the Community Steering Committee established a 5-member subcommittee with 2 academic partners (a cardiologist [L.C.B.] and a research analyst [G.B.A.]) and 3 community partners (a clergy leader [C.J.], a financial advisor [J.E.], and a community health worker [M.R.]) as a FAITH! COVID-19 Task Force. All 5 Task Force members were African American. A state-mandated stay-at-home order went into effect on March 28, 2020.

## Purpose and Objectives

The purpose of the COVID-19 response project was to promote emergency preparedness during the pandemic among African American churches. Its specific objectives were 1) to demonstrate the feasibility of implementing a COVID-19 emergency preparedness strategy within an existing CBPR partnership with local African American churches and 2) to provide local African American churches with culturally relevant, evidence-based materials via electronic communication platforms to mitigate COVID-19 risk in their communities.

## Intervention Approach

### Emergency preparedness teams

To implement an effective emergency preparedness plan, the Task Force reviewed and adapted the Centers for Disease Control and Prevention Crisis and Emergency Risk Communication (CDC-CERC) framework (13,14) in mid-March to address the COVID-19–prevention needs of an economically and socially marginalized African American faith community in Minnesota. The CDC-CERC strategy for COVID-19 control was previously tested in another CBPR partnership (15). We leveraged our ongoing ties with the network of 120 African American churches to reach populations at higher-than-average risk of acquiring or dying of COVID-19. To better understand the needs of the community, we distributed an electronic needs assessment survey by email via Qualtrics software to African American church leadership (pastors, auxiliary leaders) in our network in late March 2020. The 32 churches that responded indicated that financial support, food and utilities, and reliable COVID-19 health information were the top 3 church COVID-19 emergency resource needs. Thirty-one respondents expressed interest in receiving COVID-19 health information from FAITH! via electronic communication modalities (social media and email). Each church that completed the survey was given an American Red Cross emergency preparedness starter kit to establish a COVID-19 emergency preparedness team to serve their congregation. Aligned with the CDC-CERC framework preparation phase, we further tailored our strategy by reviewing the Humanitarian Disaster Institute’s “Preparing Your Church for Coronavirus” emergency preparedness manual (16) because of its high relevance to African American churches. In early April, we disseminated the manual to African American churches within the FAITH! network by email to assist them in establishing emergency preparedness teams.

### Training of community communication leaders

From mid-March to early April, the leadership of the Community Steering Committee trained 2 communication leaders from the Task Force as trusted messengers within the African American community. These communication leaders had the background and expertise (a community health worker and a community financial advisor) to deliver messages according to the identified church emergency resource needs. We held virtual meetings to review the intervention components and proposed messaging framework. We created a FAITH! COVID-19 Response Facebook page and an email listserv for the local African American church network as avenues to disseminate messages to the FAITH! network. Community-driven social marketing campaigns leveraging social media and email outlets have demonstrated effectiveness in raising awareness of health initiatives among African Americans while advancing social justice for the greater benefit for their communities (17,18).

### Emergency risk communication

In late March, the Task Force developed message maps to capture information on 4 main content areas: 1) inspirational messaging to promote spiritual and physical health and wellness, 2) COVID-19 health information and preventive measures consistent with Minnesota Department of Health and CDC guidelines, 3) financial and community-based resources (including food access, utilities subsidies, unemployment benefits, and testing sites) and 4) social support to foster connectedness during stay-at-home orders. Message maps are strategic tools to provide a unifying framework for disseminating information to address questions and concerns raised in a timely, accurate, and succinct manner to relevant audiences (19). Message maps facilitate effective risk communication by 1) informing and educating, 2) gaining trust and credibility, and 3) creating informed dialogue, decision making, and behavior. Information sources for message content included national and local public health agencies (eg, CDC, National Institutes of Health, Minnesota Department of Health), nonprofit community organizations (eg, The Balm in Gilead, Inc), and Mayo Clinic. We posted excerpts from the emergency preparedness manual along with encouragement to conduct church services virtually (eg, via Zoom, Facebook Live) as an alternative to in-person gatherings to maintain church engagement. Also, we developed a weekly email called the “FAITH! & COVID-19 Spread the Word Highlights of the Week!,” which summarized key messages delivered on the Facebook page with relevant attachments to allow churches to distribute this information via their email distribution lists. The communication leaders launched a social marketing campaign on April 3, 2020, via Facebook and email by posting messages daily and sending emails weekly via the African American church network email listserv. During the next few weeks, the Task Force also created culturally tailored educational resources, including infographics (10 Commandments for a Healthy Heart during the COVID-19 Pandemic), and held live videos (related to financial planning and the Coronavirus Aid, Relief, and Economic Security [CARES] Act). Furthermore, FAITH! engaged with the Association of Black Cardiologists, Inc, and advocacy groups to produce shared resources focused on counteracting widespread misinformation on COVID-19 risk within African American communities (20).

From its inception in March and throughout May, the Task Force met virtually (teleconferencing and videoconferencing) at least once per week for 60 to 90 minutes for message refinement to adapt to the rapidly evolving landscape of COVID-19 facts and resources, provide feedback on project progress, and share responses, questions, and concerns from community members.

## Evaluation Methods

We used rapid, iterative evaluation and assessment methods and a participatory evaluation approach (21). Evaluation occurred throughout intervention implementation, from April 3 to May 31, 2020, because of the continuous refinement and generation of messages to the African American church network. We collected data for process evaluation during this period. Our primary outcomes were intervention reach and engagement, feasibility, and acceptability.

### Reach and engagement

We determined emergency preparedness reach and engagement by the number of 1) church leaders who completed the needs assessment survey and received an emergency preparedness starter kit and 2) churches that received the emergency preparedness manual to start emergency preparedness teams. We determined social media and email reach and engagement by weekly tracking of Facebook participation metrics and estimating the number of people receiving the weekly highlights email communication through the African American church network email listserv. We calculated Facebook participation metrics at weekly increments by using Facebook Insights, an internal analytic software provided by Facebook (22–24). This tool is commonly used for tracking levels of reach and engagement on social networking sites, especially in the context of disseminating critical health information to racial/ethnic minority populations (22,25–27). We defined Facebook reach as the number of unique persons who viewed any particular content but did not necessarily interact with posts. We defined Facebook engagement as the number of interactions (eg, likes, loves, comments, shares, video views, post clicks) completed. We calculated total reach by summing the number of unique persons viewing content, and we calculated total engagement by summing the number of interactions per post on a weekly basis during the 8-week intervention period. We estimated email communication reach from the total number of unique persons on the African American church network email listserv (120 churches of varying congregation sizes from 50 to >1,000 members) and on each church email distribution list (ie, number of email addresses). Duplicate email addresses were removed from the email listserv. This strategy of estimating reach from a tally of email addresses from electronic mailing lists (ie, e-blasts) was used in other public health initiatives because of the challenge of measuring individual content viewing within population-level initiatives (17).

### Feasibility

To determine feasibility of the intervention as a whole, we mapped all FAITH! emergency response activities and phases to the CDC-CERC framework as advocated by CDC (13,14). We assessed feasibility and acceptability also by conducting semistructured interviews with the 2 communication leaders and reviewing summaries from weekly virtual meetings with the Task Force. Weekly Task Force meetings assessed overall satisfaction with intervention implementation by reviewing 1) weekly messages (Facebook and email), 2) message mapping, 3) educational resources design, and 4) risk communication with the African American church network. Communication leaders tracked all disseminated Facebook messages/posts and weekly emails. We conducted interviews via telephone; we audio-recorded and transcribed the interviews and de-identified participants.

### Acceptability

We conducted semistructured interviews of 15 church leaders (15 of the 32 churches that completed the needs assessment survey) using protocols that were similar to those used for the interviews of the communication leaders. The interviews assessed acceptability of our approach to 1) promote emergency preparedness within African American churches and 2) disseminate emergency risk communication within African American churches by electronic communication (Facebook and email). The interview guide was based on the CDC-CERC framework and included both open-ended and closed-ended questions. We also reviewed qualitative data on acceptability from the communication leader interviews.

### Data analyses

We summarized quantitative data for intervention reach, engagement, and acceptability as frequencies and percentages. We analyzed qualitative data on feasibility and acceptability by using integrated inductive and deductive thematic analysis (28). This process involved reading the interview transcripts in detail, generating initial coding, and identifying major themes. We then applied the interview questions as a framework to affirm the authenticity and appropriateness of the experiences evident in the raw data. We further aligned coding to the CDC-CERC framework to generate themes and subthemes and select illustrative quotes. Quantitative analyses were conducted using SPSS Statistics version 25 (IBM Corporation).

## Results

### Reach and engagement

**Figure 1:**
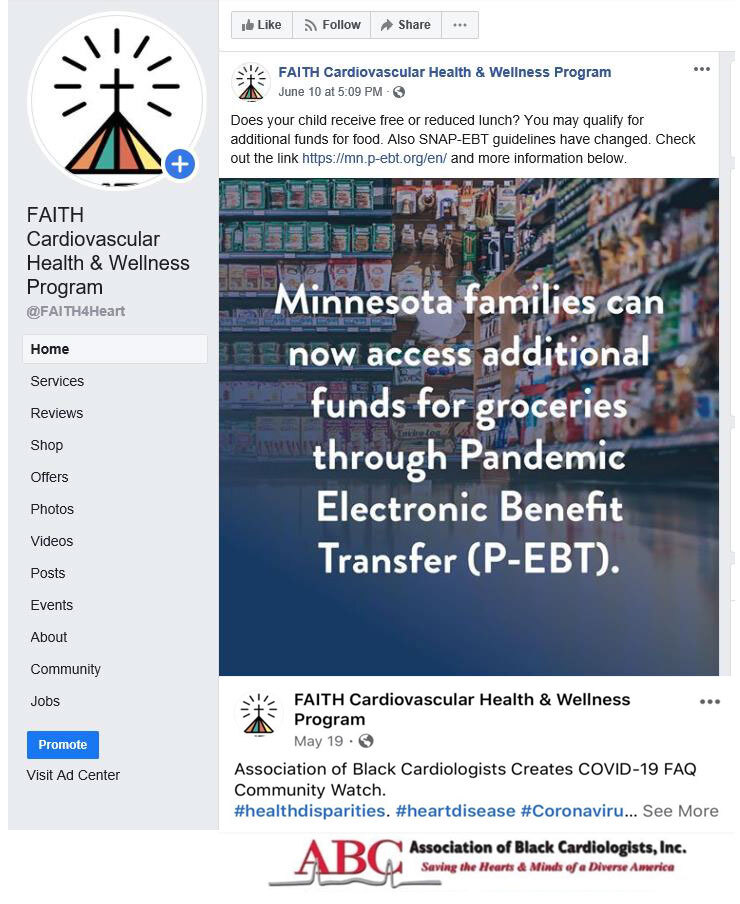
Screenshot of FAITH! (Fostering African-American Improvement in Total Health) COVID-19 response Facebook page, Minnesota, 2020. For 8 weeks (April 3–May 31, 2020), community communication leaders posted daily messages in 4 main content areas: inspirational, COVID-19 health information and preventive measures, financial and community-based resources, and social support. Content was derived from credible sources and the FAITH! COVID-19 Task Force.

**Figure 2:**
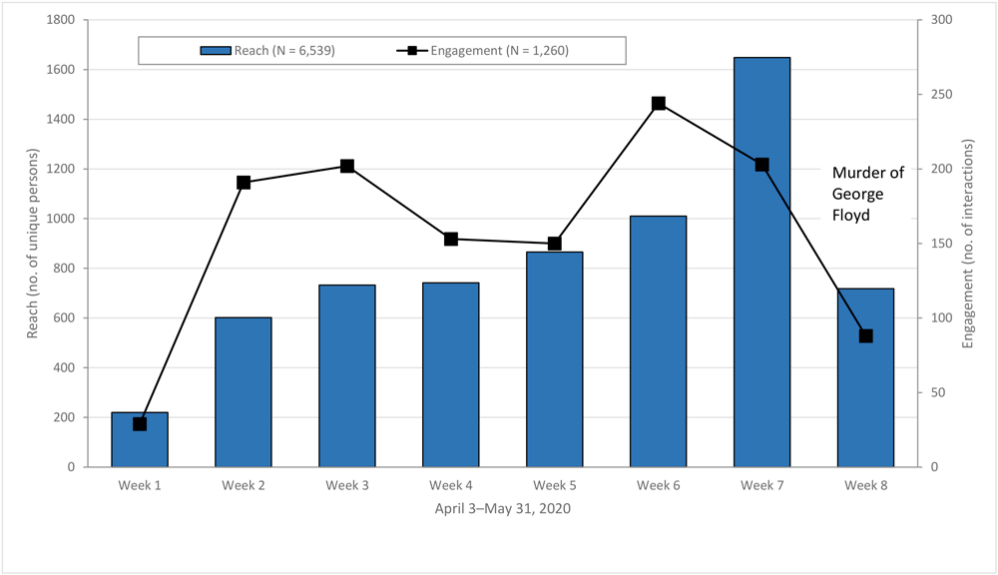
Social media (Facebook) participation metrics, FAITH! (Fostering African-American Improvement in Total Health) COVID-19 emergency risk communication, Minnesota, April 3–May 31, 2020. Reach was defined as the number of unique persons who viewed any particular content but did not necessarily interact with posts. Engagement was defined as the number of interactions (eg, likes, loves, comments, shares, video views, post clicks) completed.

We sent an invitation to complete the emergency resource needs assessment survey to 120 church leaders; 32 surveys were completed (27% completion rate). All 32 churches that completed the survey received an emergency preparedness starter kit. We distributed by email the emergency preparedness manual as a tool to start emergency preparedness to 120 African American churches, including the 32 churches that completed the survey. During the 8-week implementation period, we posted 222 messages to the Facebook page (Figure 1). These posts included photographs, infographics, links to relevant websites, polls (eg, “Does your church have an emergency preparedness team?”), and videos (eg, How to create a budget). For reach, 6,539 unique persons viewed content on the Facebook page. For engagement, we found 1,260 interactions (eg, likes, loves, comments, shares, video views, post clicks) (Figure 2). Overall, these values steadily increased during the intervention (peak at week 7 of 1,648 for reach, peak at week 6 of 244 for engagement). Reach and engagement declined precipitously on the Facebook page at week 8, which corresponded with the week of unrest that ensued after the murder of George Floyd in Minneapolis. Eight emails (1 per week) were sent to 120 churches, representing an estimated 12,000 congregation members.

**Table:** 

Week	Reach	Engagement
1	220	29
2	602	191
3	733	202
4	742	153
5	866	150
6	1,010	244
7	1,648	203
8	718	88
Total	6,539	1,260

### Feasibility

The implementation strategy using the CDC-CERC framework supports intervention feasibility (Table). The preparation phase was facilitated by organizing the emergency preparedness strategy as an extension of an established CBPR partnership with African American congregations. Conducting the needs assessment survey during this phase allowed the Task Force to best consider the prioritized needs of the churches in a sociocultural context. In the initial implementation phase, we used a collaborative approach among Task Force members to develop messages and compile culturally responsive resources for sharing by social media and email. The expertise of our communication leaders and their active communication with our population allowed us to optimize our response to the community’s needs. In the maintenance implementation phase, regular communication by virtual meetings ensured that the Task Force had a means for iterative refinement of the messaging maps and content while remaining focused on the overall project objectives to promote emergency preparedness among African American churches.

**Table:** FAITH! COVID-19 Emergency Preparedness and Risk Communication Implementation Phases in Accordance With the Centers for Disease Control and Prevention Crisis and Emergency Risk Communication Framework, 2020

Phases	Implementation Strategy
**Preparation**	
Develop partnership	• The intervention was developed as an extension of an established CBPR partnership with the African American faith community. • The FAITH! Community Steering Committee agreed on establishing a COVID-19 Task Force^a^ made up of academic and community partners to initiate an emergency response effort to the COVID-19 crisis and associated disparities in the African American community. • To better understand the needs of the prioritized population to tailor emergency risk communication, a needs assessment survey was conducted among African American churches.
Draft and test messages	• Community communication leaders on the Task Force were trained by FAITH! Community Steering Committee leadership to deliver messaging based on the needs assessment survey priorities. • Communication leaders launched a social marketing campaign via Facebook and created an email listserv for message distribution to the FAITH! church network (120 African American churches). • COVID-19 message maps were developed by the Task Force of 4 main content areas (inspirational, COVID-19 health information/preventive measures, financial and community-based resources, social support) with content from credible sources and those created by the Task Force.
Create plan	• The Task Force developed a multitiered dissemination plan at an initial virtual meeting to include supporting African American churches toward emergency preparedness through distribution of emergency preparedness starter kits and the “Preparing Your Church For Coronavirus” emergency preparedness manuals to assist with establishing emergency preparedness teams. • A plan was set to post messages daily to Facebook and send weekly highlights including content from the Facebook posts by email. • Delivering messaging through social media and email platforms would provide churches with readily accessible mediums of information to distribute to their congregation members. The messaging plan was refined weekly by the Task Force at virtual meetings.
Determine approval process	• All messages were reviewed and approved by Task Force members before distribution on social media or by email. • Messages were cross-referenced and updated daily with CDC and local health department content.
**Initial**	
Explain risk	• Messages emphasized strategies to mitigate the spread and transmission of COVID-19. • A focus was placed on reaching high-risk populations, including essential workers in service-sector jobs, the elderly, and those with chronic medical conditions. • Messages also focused on dispelling myths about lower perceived COVID-19 risk among African Americans.
Promote action	• Most messages were action-oriented with a focus on COVID-19 prevention strategies, testing, and available community-based resources. • There was also an emphasis on church emergency preparedness with excerpts from the *Preparing Your Church for Coronavirus* emergency preparedness manual and promotion of use of virtual church services (eg, Zoom, Facebook Live) to maintain church engagement. • Network churches’ COVID-19 response efforts were also highlighted to create an information hub of available resources. Messages were refined regularly and tailored in response to community members’ needs and questions.
Describe response efforts (establish organization’s credibility)	• The established infrastructure and credibility of the FAITH! academic–community partnership facilitated rapid implementation of the response efforts. • The Task Force had weekly virtual meetings, which allowed for bidirectional communication about the real-time effect of the response and community member feedback. • In addition to the virtual meetings, communication leaders maintained contact with each other and the Task Force through emails and text messages. • Communication leaders also maintained and updated the FAITH! Facebook page and email listserv to disseminate the daily messages. • This participatory approach promoted ownership of the intervention by communication leaders and contributed to the intervention’s success.
**Maintenance**	
Provide background information	• The underlying theme of messaging throughout the maintenance phase was COVID-19 prevention, including basic strategies of defense, such as hand washing, wearing facemasks, social/physical distancing, and following stay-at-home mandates. Information on available testing sites were also included.
Explain ongoing risks	• Given the increased risk of acquiring COVID-19 among the African American community, messages were posted in the sociocultural context to explain underlying etiologies of this higher susceptibility. These include preexisting comorbidities (cardiovascular disease and its risk factors, hypertension, diabetes, obesity), structural/institutional inequities (less access to care/testing sites, densely populated residential housing), and socioeconomic challenges (financial, inability to stay at home as essential workers, etc). • Regional, state, and national COVID-19 infection rates, including number of deaths by race, were also disseminated to reinforce the message of ongoing risk among African Americans and other racial/ethnic minority groups.
Segment audiences	• Messages were adapted for church networks in each region (Rochester and Minneapolis–St. Paul, Minnesota) to increase awareness of available resources in each area. Messages were culturally tailored to the needs and preferences of the African American faith community.
Address rumors	• Rumors or myths about COVID-19 risk and prevention were compiled by the communication leaders from interaction with community members on social media, email, and their networks. Frequent rumors encountered included home remedies for COVID-19 prevention and treatment, treatment efficacy (hydroxychloroquine), vaccine availability, and risk misperception. • Rumors and myths were discussed and directly addressed during weekly virtual meetings and in real time by the communication leaders with the Task Force, in particular FAITH! Community Steering Committee leadership. • Materials were created and disseminated to dispel rumors and myths surrounding COVID-19.
**Resolution**	
Motivate vigilance	• Maintaining a consistent line of bidirectional communication with the church networks through social media and email aimed to maintain vigilance. • Communication leader participation fatigue and complacency were counteracted through regular Task Force meetings, positive reinforcement by FAITH! Community Steering Committee leadership, and through adherence to CBPR principles that place value on equal ownership of the response effort by community and academic partners.
Discuss, document lessons learned	• Lessons learned were compiled from 3 sources: semistructured interviews with communication leaders and African American church network leaders as well as through weekly Task Force virtual meetings. • Transparency among Task Force members and process evaluation allowed for rapid refinement of the implementation plan and messaging to simultaneously meet the needs of communication leaders and the African American church network. • Review of documented lessons learned allowed for team building, continuous improvement, and allocation of institutional resources to further support the project.
Evaluate and revise plans	• Rapid evaluation led to several revised implementation plans including more direct instructions on the purpose and proper use of the emergency preparedness manual to establish effective church emergency preparedness teams, more messages promoting relevant webinars and town halls on COVID-19 in the African American community, and specific messages on childcare and school reopening. • Next steps include providing state and CDC-based guidelines and resources for safe church reopening.

Abbreviations: CBPR, community-based participatory research; CDC, Centers for Disease Control and Prevention; COVID-19, coronavirus disease 2019; FAITH, Fostering African-American Improvement in Total Health.

^a^ Task Force members included community and academic leaders, communication leaders, other volunteers, and representatives from regional community-based organizations.

Furthermore, the intervention emergency risk communication was successfully implemented by the 2 communication leaders during the 8-week monitoring period. We held 8 weekly virtual meetings to ensure responsiveness to the local COVID-19 efforts and community member concerns. Emerging themes noted by the 2 communication leaders attested to the feasibility of the intervention as a culturally relevant, essential response effort to mitigate spread of COVID-19 in the African American community:

The experience I’ve had as a communication leader has been rewarding. I enjoy finding up-to-date, relevant information on COVID-19 and providing resources specifically tailored to the African-American community. [Community health worker]

Both communication leaders found a sense of empowerment and gratification in leading an initiative to address the pandemic’s negative effect on African Americans:

The position of Communication Leader gives me a chance to serve the community. I believe serving others blesses me in return. It warms my heart to know I’m able to give my time to a program that is needed and appreciated by community members. It is my passion to give back to the community and I am grateful for this opportunity. [Community health worker]

If our community can stay in the know on resources, credible information and events we are making an impact. Our goal is to make sure that we are a trusted resource for COVID-19 and health-related topics that impact communities of color, specifically communities of faith. I have enjoyed being a part of this engagement. [Community financial advisor]

They found the bidirectional communication with the African American churches via social media and email useful for real-time enhancement of messaging.

### Acceptability

Church leaders had overall positive perceptions of the FAITH! COVID-19 emergency response efforts. Eleven of the 15 church leaders interviewed reported that the outreach and information from FAITH! improved their church’s emergency preparedness levels. Furthermore, they indicated that FAITH! was a trusted and credible information source for dissemination of health and community-based information to this population:

I guess the one thing that stands out is how the FAITH! Program has gathered all this information and put it into an email form or through their website [Facebook page], and just made it available . . . but the type of information that has been given out has really been impactful. [Age 69, female, trustee chair]

Because the congregants know that the FAITH! project is tailored toward the African American community, one of the things is it’s reliable. It’s believed to be reliable and trustworthy, so that’s the primary thing. It’s coming from a source that, you know, I can trust this. [Age 73, female, president of Health and Social Justice Ministry]

Sentiments about the effect of the intervention on the African American faith community were also corroborated by the communication leaders:

Providing local resources for services such as access to food and essentials, rent and utility assistance, free COVID-19 testing sites through daily Facebook posts and weekly emails has provided church leaders additional information to provide to congregants in need. It takes the strain off of the church to have to find the resources, distribute them, and provide support throughout the process. With information on COVID-19 changing rapidly and the various daily updates it can be hard for community members to keep track. I believe providing COVID-19 updates from reliable sources has made it easier for community members to navigate and follow the updated guidelines. [Community health worker]

People are using our page and community/church leaders are also interacting in such a way as to provide us with events that they have coming up to pass along to the community. We bring attention to all of the areas that have been impacted by this pandemic. This well-rounded approach is key to supporting our community in a more holistic way. [Community financial advisor]

The time commitment involved by the communication leaders was deemed reasonable and not overwhelming:

The requirement of posting the information is flexible, so I can easily work it into my schedule. Some of the social media posts can be set to go out days ahead of time. This feature helps me when I have other deadlines to meet or prior commitments that particular day. [Community health worker]

Daily posts are not time-intensive. The scheduling features on Facebook allow us to plan ahead with some information. The COVID-19 specific postings are usually posted day of as we want to keep the information as up-to-date and relevant as possible. [Community financial advisor]

In addition, the church leaders identified the top 3 most useful and reliable sources of information related to COVID-19 as FAITH!/Mayo Clinic (n = 8), briefings by the Minnesota Governor (n = 8), and briefings by CDC (n = 7). All 15 church leaders expressed their desire to continue to receive COVID-19 health information from FAITH! for dissemination to their church members during the pandemic.

## Implications for Public Health

Given the COVID-19 disparities among African Americans, community-based pandemic emergency response efforts are essential to serve and protect the health and well-being of this population. Leveraging the existing infrastructure of an academic–community partnership to support this response facilitates rapid dissemination of culturally relevant resources and materials to economically and socially marginalized communities whose socioeconomic challenges are exacerbated by a public health crisis. Identifying emergency resource needs and quickly implementing COVID-19 emergency preparedness strategies within embedded trusted institutions in these communities can equip them to safely navigate the pandemic. In turn, these institutions can serve as conduits to connect communities to accurate COVID-19–related health information and critical resources to mitigate and reverse COVID-19–related inequities.

Our mobilization of trusted community messengers, including a community health worker, and strategic use of readily accessible and actively used technologies, such as Facebook, by African Americans (29) further strengthened our efforts and can be replicated in other economically and socially marginalized communities. Some surveys report that African Americans trust information in social media platforms such as Facebook for COVID-19 health information more than other racial/ethnic groups, likely because they rely on these channels for news and activism (30,31).

We recognize that a key limitation to our process evaluation was the lack of inclusion of measures of effect on individual behavior change in COVID-19 preventive measures as a direct result of viewing our intervention materials. However, follow-up formative evaluation may provide insights on the influence of our intervention on church congregation members. Nevertheless, our intervention fulfilled its principal purpose of fostering public engagement and partnerships to rapidly promote COVID-19 emergency preparedness and risk communication among African American churches in a relatively short period. As the COVID-19 pandemic continues, similar strategies will be warranted to provide further community and population-level risk mitigation and to promote vaccine receptivity among racial/ethnic minority groups. The CBPR approach employed by FAITH! can serve as a model for others planning to design and implement campaigns and initiatives to address the health concerns of populations at higher-than-average risk of acquiring or dying of COVID-19.

Similar COVID-19 awareness programs, including webinars (32), teleconference calls (33), and virtual town halls (34), within established academic–community coalitions have also demonstrated success in outreach via electronic communication platforms to under-resourced communities. Central to the success of these partnerships is the embedded value of placing a priority on transforming academic institutions into trustworthy vehicles of change (35). Such efforts by partner academic institutions demonstrate a sincere commitment to economically and socially marginalized communities and help to build trust that the programs and interventions will ensure the safety and well-being of those communities during a time of crisis.

## References

[1] Blevins JB , Jalloh MF , Robinson DA . Faith and global health practice in Ebola and HIV emergencies. Am J Public Health 2019;109(3):379–84. 10.2105/AJPH.2018.304870 30676797 PMC6366492

[2] Kiser M , Lovelace K . A national network of public health and faith-based organizations to increase influenza prevention among hard-to-reach populations. Am J Public Health 2019;109(3):371–7. 10.2105/AJPH.2018.304826 30676795 PMC6366510

[3] Haynes N , Cooper LA , Albert MA ; Association of Black Cardiologists. At the heart of the matter: unmasking and addressing COVID-19’s toll on diverse populations. Circulation 2020;142(2):105–7. 10.1161/CIRCULATIONAHA.120.048126 32364762

[4] Cooper LA , Williams DR . Excess deaths from COVID-19, community bereavement, and restorative justice for communities of color. JAMA 2020;324(15):1491–2. 10.1001/jama.2020.19567 33044518

[5] Yancy CW . COVID-19 and African Americans. JAMA 2020;323(19):1891–2. 10.1001/jama.2020.6548 32293639

[6] Pew Research Center. Health concerns from COVID-19 much higher among Hispanics and Blacks than Whites. 2020. https://www.pewresearch.org/politics/2020/04/14/health-concerns-from-covid-19-much-higher-among-hispanics-and-blacks-than-whites. Accessed August 5, 2020.

[7] Brewer LC , Williams DR . We’ve come this far by faith: the role of the Black church in public health. Am J Public Health 2019;109(3):385–6. 10.2105/AJPH.2018.304939 30726121 PMC6366503

[8] Pullins CT , Seele PC , White RO , Willis FB , Poole K , Albertie ML , Health behaviors and preventive healthcare utilization among African-American attendees at a faith-based public health conference: Healthy Churches 2020. J Relig Health 2018;57(6):2538–51. 10.1007/s10943-018-0667-2 29995232 PMC7249222

[9] Giger JN , Appel SJ , Davidhizar R , Davis C . Church and spirituality in the lives of the African American community. J Transcult Nurs 2008;19(4):375–83. 10.1177/1043659608322502 18650398

[10] Manjunath C , Ifelayo O , Jones C , Washington M , Shanedling S , Williams J , Addressing cardiovascular health disparities in Minnesota: establishment of a community steering committee by FAITH! (Fostering African-American Improvement in Total Health). Int J Environ Res Public Health 2019;16(21):E4144. 10.3390/ijerph16214144 31661826 PMC6862476

[11] Brewer LC , Hayes SN , Caron AR , Derby DA , Breutzman NS , Wicks A , Promoting cardiovascular health and wellness among African-Americans: community participatory approach to design an innovative mobile-health intervention. PLoS One 2019;14(8):e0218724. 10.1371/journal.pone.0218724 31430294 PMC6701808

[12] Minnesota Department of Health. Health officials confirm first case of novel coronavirus in Minnesota. 2020. https://www.health.state.mn.us/news/pressrel/2020/covid19030620.html. Accessed August 2, 2020.

[13] Centers for Disease Control and Prevention. Crisis and emergency risk communication manual. 2018. https://emergency.cdc.gov/cerc/manual/index.asp . Accessed October 2, 2020.

[14] Centers for Disease Control and Prevention. 2018. Crisis and emergency risk communication manual, CERC: community engagement. https://emergency.cdc.gov/cerc/ppt/CERC_CommunityEngagement.pdf. Accessed October 2, 2020.

[15] Wieland ML , Asiedu GB , Lantz K , Abbenyi A , Njeru JW , Osman A , Leveraging community engaged research partnerships for crisis and emergency risk communication to vulnerable populations in the COVID-19 pandemic. J Clin Transl Sci 2020:1–5. 10.1017/cts.2020.47 33942018 PMC7605400

[16] Humanitarian Disaster Institute. Preparing your church for coronavirus. 2020. https://www.health.state.mn.us/diseases/coronavirus/guidefaith.pdf. Accessed July 26, 2020.

[17] George KS , Roberts CB , Beasley S , Fox M , Rashied-Henry K ; Brooklyn Partnership to Drive Down Diabetes (BP3D). Our health is in our hands: a social marketing campaign to combat obesity and diabetes. Am J Health Promot 2016;30(4):283–6. 10.1177/0890117116639559 27404065

[18] Merchant RM , Lurie N . Social media and emergency preparedness in response to novel coronavirus. JAMA 2020;323(20):2011–2. 10.1001/jama.2020.4469 32202611

[19] Covello VT . Risk communication and message mapping: a new tool for communicating effectively in public health emergencies and disasters. J Emerg Manag 2006;4(3):25–40. 10.5055/jem.2006.0030

[20] Cardiovascular Business. Association of Black Cardiologists shares COVID-19 resources for patients, providers. 2020. https://www.cardiovascularbusiness.com/topics/practice-management/association-black-cardiologists-covid-19. Accessed August 16, 2020.

[21] McNall M , Foster-Fishman PG . Methods of rapid evaluation, assessment, and appraisal. Am J Eval 2007;28(2):151–68. 10.1177/1098214007300895

[22] Neiger BL , Thackeray R , Van Wagenen SA , Hanson CL , West JH , Barnes MD , Use of social media in health promotion: purposes, key performance indicators, and evaluation metrics. Health Promot Pract 2012;13(2):159–64. 10.1177/1524839911433467 22382491

[23] Zanon M , Altmayer S , Pacini GS , Guedes Á , Watte G , Marchiori E , Facebook as a tool to promote radiology education: expanding from a local community of medical students to all of South America. Radiol Bras 2018;51(4):242–7. 10.1590/0100-3984.2017.0112 30202128 PMC6124594

[24] Facebook for Business. An update on metrics and reporting. 2016. https://www.facebook.com/business/news/metrics-reporting-update. Accessed October 2, 2020.

[25] Gamboa J , Lamb MM , de la Cruz P , Bull S , Olson D . Using social media to increase preventative behaviors against arboviral diseases: a pilot study among teens in the Dominican Republic. mHealth 2019;5:30. 10.21037/mhealth.2019.07.03 31559275 PMC6737439

[26] Klassen KM , Borleis ES , Brennan L , Reid M , McCaffrey TA , Lim MS . What people “like”: analysis of social media strategies used by food industry brands, lifestyle brands, and health promotion organizations on Facebook and Instagram. J Med Internet Res 2018;20(6):e10227. 10.2196/10227 29903694 PMC6024098

[27] Sesagiri Raamkumar A , Tan SG , Wee HL . Measuring the outreach efforts of public health authorities and the public response on Facebook during the COVID-19 pandemic in early 2020: cross-country comparison. J Med Internet Res 2020;22(5):e19334. 10.2196/19334 32401219 PMC7238862

[28] Fereday J , Muir-Cochrane E . Demonstrating rigor using thematic analysis: a hybrid approach of inductive and deductive coding and theme development. Int J Qual Methods 2006;5(1):80–92. 10.1177/160940690600500107

[29] Pew Research Center. Social media fact sheet. 2019. https://www.pewresearch.org/internet/fact-sheet/social-media/#who-uses-each-social-media-platform . Accessed October 2, 2020.

[30] Pew Research Center. Activism on social media varies by race and ethnicity, age, political party. 2020. https://www.pewresearch.org/fact-tank/2020/07/13/activism-on-social-media-varies-by-race-and-ethnicity-age-political-party. Accessed October 2, 2020.

[31] Capital Public Radio. African Americans more likely to trust Social media for COVID information. 2020. https://www.capradio.org/articles/2020/07/21/african-americans-more-likely-to-trust-social-media-for-covid-information. Accessed October 2, 2020.

[32] Calo WA , Murray A , Francis E , Bermudez M , Kraschnewski J . Reaching the Hispanic community about COVID-19 through existing chronic disease prevention programs. Prev Chronic Dis 2020;17:E49. 10.5888/pcd17.200165 32584753 PMC7316420

[33] Galiatsatos P , Monson K , Oluyinka M , Negro D , Hughes N , Maydan D , community calls: lessons and insights gained from a medical-religious community engagement during the COVID-19 pandemic. J Relig Health 2020;59(5):2256–62. 10.1007/s10943-020-01057-w 32594340 PMC7320249

[34] Fletcher FE , Allen S , Vickers SM , Beavers T , Hamlin CM , Young-Foster D , COVID-19’s impact on the African American community: a stakeholder engagement approach to increase public awareness through virtual town halls. Health Equity 2020;4(1):320–5. 10.1089/heq.2020.0029 32775941 PMC7410280

[35] Warren RC , Forrow L , Hodge DA Sr , Truog RD . Trustworthiness before Trust — Covid-19 vaccine trials and the Black community. N Engl J Med 2020;NEJMp2030033. 10.1056/NEJMp2030033 33064382

